# Association of serum uric acid with hepatic steatosis detected by controlled attenuation parameter in the United States population

**DOI:** 10.1186/s12944-023-01846-8

**Published:** 2023-06-20

**Authors:** Yunfu Feng, Sijie Zheng, Luojie Liu, Yanting Yang

**Affiliations:** 1 Endoscopy Center, The First People’s Hospital of Kunshan, Kunshan, 215300 China; 2 Department of Gastroenterology, The Third People’s Hospital of Kunshan, Kunshan, 215300 China; 3 Department of Gastroenterology, Changshu Hospital Affiliated to Soochow University, Changshu, 215500 China

**Keywords:** Serum uric acid, Nonalcoholic fatty liver disease, Controlled attenuation parameter, NHANES, A cross-sectional study

## Abstract

**Background:**

The relationship between serum uric acid (SUA) and nonalcoholic fatty liver disease (NAFLD) has been previously reported. Controlled attenuation parameter (CAP) has better diagnostic performance than ultrasonography for assessing hepatic steatosis. The association of SUA with hepatic steatosis detected by CAP is worth further study.

**Methods:**

The US population aged 20 years or older from the National Health and Nutrition Examination Survey (NHANES) was assessed. Hepatic steatosis was evaluated by the controlled attenuation parameter (CAP). NAFLD status was defined as CAP values of 268 dB/m without hepatitis B or C virus infection or considerable alcohol consumption. Multiple imputations were performed to fill in the missing covariate values. Linear regression, logistic regression, and smooth curve fitting were used to examine the association.

**Results:**

In total, 3919 individuals participated in this study. There was a positive association between SUA (µmol/L) and CAP (β = 0.14, 95% CI: 0.12-0.17, *P* < 0.01). After stratification by sex, a significant relationship between SUA and CAP existed in both males (β = 0.12, 95% CI: 0.09-0.16, *P* < 0.01) and females (β = 0.17, 95% CI: 0.14-0.20, *P* < 0.01) after multiple imputation. The inflection points of the threshold effect of SUA on CAP were 487.7 µmol/L in males and 386.6 µmol/L in females. There was a positive association between SUA (mg/dL) and NAFLD (OR = 1.30, 95% CI: 1.23-1.37, *P* < 0.01). After stratification by race, positive relationships were also observed. Meanwhile, a positive relationship existed between hyperuricemia and NAFLD (OR = 1.94, 95% CI: 1.64-2.30, *P* < 0.01). The positive relationship was more significant in females than in males (*P* for interaction < 0.01).

**Conclusions:**

There was a positive association between SUA and CAP, as well as between SUA and NAFLD. Subgroup studies stratified by sex and ethnicity demonstrated that the effects were consistent.

**Supplementary Information:**

The online version contains supplementary material available at 10.1186/s12944-023-01846-8.

## Introduction

Nonalcoholic fatty liver disease (NAFLD) is the most common cause of chronic liver disease worldwide [1]. NAFLD is a disease spectrum defined by hepatic steatosis (HS), with epidemiological statistics indicating a global prevalence of 25% [2–4]. NAFLD is characterized by lipid accumulation, insulin resistance, and metabolic stress-induced liver injury [5, 6]. Obesity, type 2 diabetes, hyperlipidemia, hypertension, and metabolic syndrome are all frequently related to NAFLD [4, 7]. Patients with NAFLD can be assessed in a variety of ways, including ultrasonography, magnetic resonance imaging (MRI), vibration controlled transient elastography (VCTE) and hepatic biopsy [8, 9]. Hepatic biopsy is regarded as the gold standard for determining the severity of HS [10]. However, hepatic biopsy is an invasive procedure not available for large-scale screenings. Noninvasive approaches for assessing HS have been developed. Among imaging approaches, the controlled attenuation parameter (CAP) function of the FibroScan device appears to be the most promising noninvasive test for quantifying HS [11].

Serum uric acid (SUA) is the last product of purine metabolism, acting as a natural antioxidant in humans. A rise or fall in SUA levels can cause or disclose a variety of disorders [12]. Hyperuricemia, similar to NAFLD, is intimately associated with metabolic dysregulation, such as obesity and insulin resistance (IR) [13, 14]. Several pieces of evidence suggest that increased SUA plays a crucial role in the pathophysiology of NAFLD [15, 16]. However, in different sexes, the link between SUA and NAFLD has proven to be disputed. Recent research on 541 women found that SUA was not independently related to the risk of NAFLD [17]. A beneficial relationship between SUA and NAFLD was found in females but not in males [18]. Moreover, previous studies rarely evaluated ethnic subgroups.

Hence, we conducted a cross-sectional study to investigate the association between SUA and hepatic steatosis detected by CAP in the US population from the 2017–2018 National Health and Nutrition Examination Survey (NHANES). A further detailed evaluation by sex and ethnicity was also performed.

## Methods

### Study population

The National Health and Nutrition Examination Survey (NHANES) is a cross-sectional research project managed by the Centers for Disease Control and Prevention (CDC) that examines the health status of the nationwide population. The National Center for Health Statistics (NCHS) ethics review board approved the study, and all individuals signed an informed consent form.

These data are available on the website of NHANES 2017–2018 (available online at: http://www.cdc.gov/NCHS/nhanes.htm). After excluding age < 20 years (N = 3685), missing transient elastography (N = 1059), missing SUA (N = 244), individuals with hepatitis B (N = 15), hepatitis C (N = 22), and considerable alcohol consumption (≥ 30 g for males and ≥ 20 g for females) (N = 310), 3919 individuals took part in this study in total.

### Variables

The exposure variable was serum uric acid, which was measured by the Roche Cobas 6000 Chemistry Analyzer. Uric acid is oxidized by uricase in this approach. The peroxide formed by this reaction is then acted on by peroxidase in the presence of 4 aminophenazone to yield a detectable colored product. It is a two-point, endpoint reaction, with the measurement taking place at 546 nm. SUA values were displayed in µmol/L, which were translated to mg/dL by dividing by 59.5. Hyperuricemia was defined as SUA concentrations ≥ 420 µmol/L in males and ≥ 360 µmol/L in females [19].

The outcome variable was hepatic steatosis detected by CAP using FibroScan performed on eligible participants. NAFLD status was defined as CAP values of 268 dB/m without hepatitis B or C virus infection or considerable alcohol consumption. Based on the AUROC values (AUROC 0.86) and commonly used cutoff point, 268 dB/m was defined as the cutoff point in our study. Examinations were considered reliable if they had a fasting time of at least 3 h and 10 or more complete measures.

### Covariates

The continuous covariates were age, body mass index (BMI), waist circumference, ratio of family income, alanine aminotransferase (ALT), aspartate aminotransferase (AST), serum creatinine, gamma glutamyl transferase (GGT), serum cholesterol, triglycerides, high-density lipoprotein cholesterol (HDL-C), low-density lipoprotein cholesterol (LDL-C), glycohemoglobin, HOMA-IR, dietary fat intake, and physical activity. The category covariates were gender, race/ethnicity, education levels, smoking habits, diabetes, and hypertension.

The demographic questionnaires for the family and sample members were administered in the house by trained interviewers utilizing the Computer Assisted Personal Interview (CAPI) system, including age, gender, ethnicity, education levels, and ratio of family income. The blood samples were forwarded to the NHANES laboratory for analysis, including liver and kidney function, blood lipids, and glycohemoglobin. The formula HOMA-IR = fasting insulin (mU/L) * fasting glucose (mmol/L)/22.5 was used to calculate HOMA-IR. Physical activity was determined by multiplying the weekly amount of time spent in each activity by the metabolic equivalent of the task (MET). Data on dietary fat intake were obtained from the 24-hour dietary survey. Ethnicity was classified as non-Hispanic white, non-Hispanic black, Mexican American, other Hispanic and other race. The levels of education were less than 12th grade, high school graduate or college degree, and college graduate or above. Smoking habits were classified as current, former, or never. Hypertension and diabetes were assessed by self-report.

### Statistical analyses

The continuity variables were recorded as the mean ± standard deviation (SD), and the categorical variables were recorded as percentages. The missing values of covariates were treated through the multiple imputation procedure. Multiple imputations were used in our analysis, based on five replications and a chained equation approach method in the R MI procedure. Because parts of the covariates were not normally distributed, log e transformations were conducted for analysis. Multivariate linear regression models were applied to assess the association between SUA and CAP. Multivariate logistic regression models were applied to assess the association between SUA and NAFLD. Age, gender, race, BMI, waist circumference, education levels, ratio of family income, ALT, AST, GGT, serum creatinine, triglyceride, serum cholesterol, HDL-C, LDL-C, glycohemoglobin, HOMA-IR, dietary fat intake, smoking habits, MET, hypertension, and diabetes were considered potential factors. To address the nonlinear relationship, smooth curve fitting and a generalized additive model were used. The threshold effect of the nonlinear relationship between SUA and CAP was investigated using a segmented regression model. A statistically significant difference was shown by a two-sided *P* < 0.05. All statistical data were analyzed using R (http://www.R-project.org, The R Foundation, Boston, MA, USA) and EmpowerStats software (http://www.empowerstats.com, X&Y Solutions, Inc., Boston, MA, USA).

## Results

An overview of the general characteristics of the study subjects is shown in Table 1, including age, gender, race/ethnicity, education levels, ratio of family income, BMI, waist circumference, ALT, AST, GGT, serum creatinine, triglyceride, serum cholesterol, HDL-C, LDL-C, glycohemoglobin, HOMA-IR, dietary fat intake, smoking habits, MET, smoking habits, hypertension, and diabetes. Our study enrolled 3919 individuals, including 1971 males and 1948 females. The mean SUA was 325.98 ± 88.35 µmol/L. The prevalence of hyperuricemia was 19.32% in total, 21.87% in males, and 16.74% in females. The mean median CAP was 266.32 ± 62.23 dB/m.

**Table 1:** Characteristics of the study population

General characteristics		Missing N	multiple imputations
Age (years)	51.14 ± 17.31	0	51.14 ± 17.31
Males (%)	50.29	0	50.29
Race/Ethnicity (%)		0	
Non-Hispanic White	32.61		32.61
Non-Hispanic Black	23.65		23.65
Mexican American	15.03		15.03
Other Hispanic	8.91		8.91
Other Race	19.80		19.80
Education levels (%)		1591	
Less than 12th grade	18.72		25.26
High school graduate or college degree	56.49		54.88
College graduate or above	24.79		19.86
Ratio of family income	2.40 ± 1.60	536	0.58 ± 0.91*
BMI	26.49 ± 8.35	498	3.20 ± 0.31*
Waist circumference (cm)	89.50 ± 22.73	684	4.47 ± 0.27*
ALT (U/L)	21.07 ± 16.69	1391	2.88 ± 0.55*
AST (U/L)	21.69 ± 13.57	1391	2.96 ± 0.37*
GGT (U/L)	30.07 ± 50.30	1391	2.99 ± 0.71*
Serum creatinine (mmol/L)	76.69 ± 36.73	1390	4.31 ± 0.30*
Triglyceride (mmol/L)	1.23 ± 1.27	1392	0.04 ± 0.61*
Serum cholesterol (mmol/L)	4.74 ± 1.08	1390	4.75 ± 1.07
HDL-C (mmol/L)	1.39 ± 0.38	990	1.37 ± 0.38
LDL-C (mmol/L)	2.80 ± 0.93	1203	0.98 ± 0.34*
Glycohemoglobin (%)	5.76 ± 0.99	1335	5.71 ± 0.96
HOMA-IR	3.61 ± 5.42	2657	0.92 ± 0.76*
Dietary fat intake (g)	80.74 ± 48.43	710	4.17 ± 0.66*
Physical activity (MET)	699.61 ± 2768.99	542	6.52 ± 1.31*
Smoking habits (%)		1460	
current	17.81		14.79
former	21.47		18.94
never	60.72		66.27
Hypertension (%)	33.66	1331	27.61
Diabetes (%)	9.66	224	9.61
*Exposures*			
SUA (µmol/L)	325.98 ± 88.35	0	/
SUA (mg/dL)	5.48 ± 1.49	0	/
Hyperuricemia (%)	19.32	0	/
*Outcomes*			
Median CAP (dB/m)	266.32 ± 62.23	0	/

*log_e_-transformed

BMI: body mass index, ALT: alanine aminotransferase, AST: aspartate aminotransferase, GGT: gamma glutamyl transferase, HDL-C: high-density lipoprotein cholesterol, LDL-C: low-density lipoprotein cholesterol, HOMA-IR: fasting insulin (mU/L) * fasting glucose (mmol/L)/22.5, CAP: controlled attenuation parameter, SUA: serum uric acid

Table 2 displays the outcomes of the multivariate linear regression. There was a significant association between SUA and median CAP in all models, with or without multiple imputations. Stratified by sex, a positive relationship between SUA and CAP existed in both males (β = 0.12, 95% CI: 0.09-0.16, *P* < 0.01) and females (β = 0.17, 95% CI: 0.14-0.20, *P* < 0.01) after multiple imputation. The correlation between SUA and CAP in males in model 2 (β = 0.08, 95% CI: −0.01-0.16, *P* = 0.06) was negligible before imputations. After stratification by race, as shown in Supplementary Table S1, positive relationships were also observed. Similarly, the outcomes for hyperuricemia and CAP were the same.

**Table 2:** Association between SUA (µmol/L) and CAP among US adults aged ≥ 20 years

	Crude	*P* values	Model 1	*P* values	Model 2	*P* values	Model 1^a^	*P* values	Model 2^a^	*P* values
SUA	0.17 (0.15, 0.20)	< 0.01	0.16 (0.13, 0.18)	< 0.01	0.14 (0.08, 0.20)	< 0.01	0.16 (0.14, 0.19)	< 0.01	0.14 (0.12, 0.17)	< 0.01
Male	0.14 (0.10, 0.17)	< 0.01	0.13 (0.10, 0.17)	< 0.01	0.08 (–0.01, 0.16)	0.06	0.14 (0.10, 0.17)	< 0.01	0.12 (0.09, 0.16)	< 0.01
Female	0.22 (0.18, 0.25)	< 0.01	0.18 (0.15, 0.22)	< 0.01	0.22 (0.13, 0.31)	< 0.01	0.19 (0.16, 0.23)	< 0.01	0.17 (0.14, 0.20)	< 0.01
Hyperuricemia	29.18 (24.36, 34.00)	< 0.01	27.57 (22.46, 32.67)	< 0.01	24.02 (11.09, 36.96)	< 0.01	27.27(22.84, 32.56)	< 0.01	23.60(19.30, 28.86)	< 0.01
Male	24.77 (18.08, 31.46)	< 0.01	25.74 (18.70, 32.78)	< 0.01	15.64 (–2.98, 34.26)	0.09	24.67(18.73, 32.51)	< 0.01	22.19(16.42, 29.99)	< 0.01
Female	34.66 (27.70, 41.61)	< 0.01	29.11 (21.57, 36.64)	< 0.01	32.72 (13.08, 52.35)	< 0.01	30.07(23.72, 38.12)	< 0.01	25.20(18.96, 33.50)	< 0.01

^a^ Missing values of covariates were imputed with multiple imputations

Model 1: age, race and BMI were adjusted

Model 2: age, race, BMI, waist circumference, education levels, ratio of family income, ALT, AST, GGT, serum creatinine, triglyceride, serum cholesterol, HDL-C, LDL-C, glycohemoglobin, HOMA-IR, dietary fat intake, smoking habits, MET, hypertension and diabetes were adjusted

**Fig. 1:**
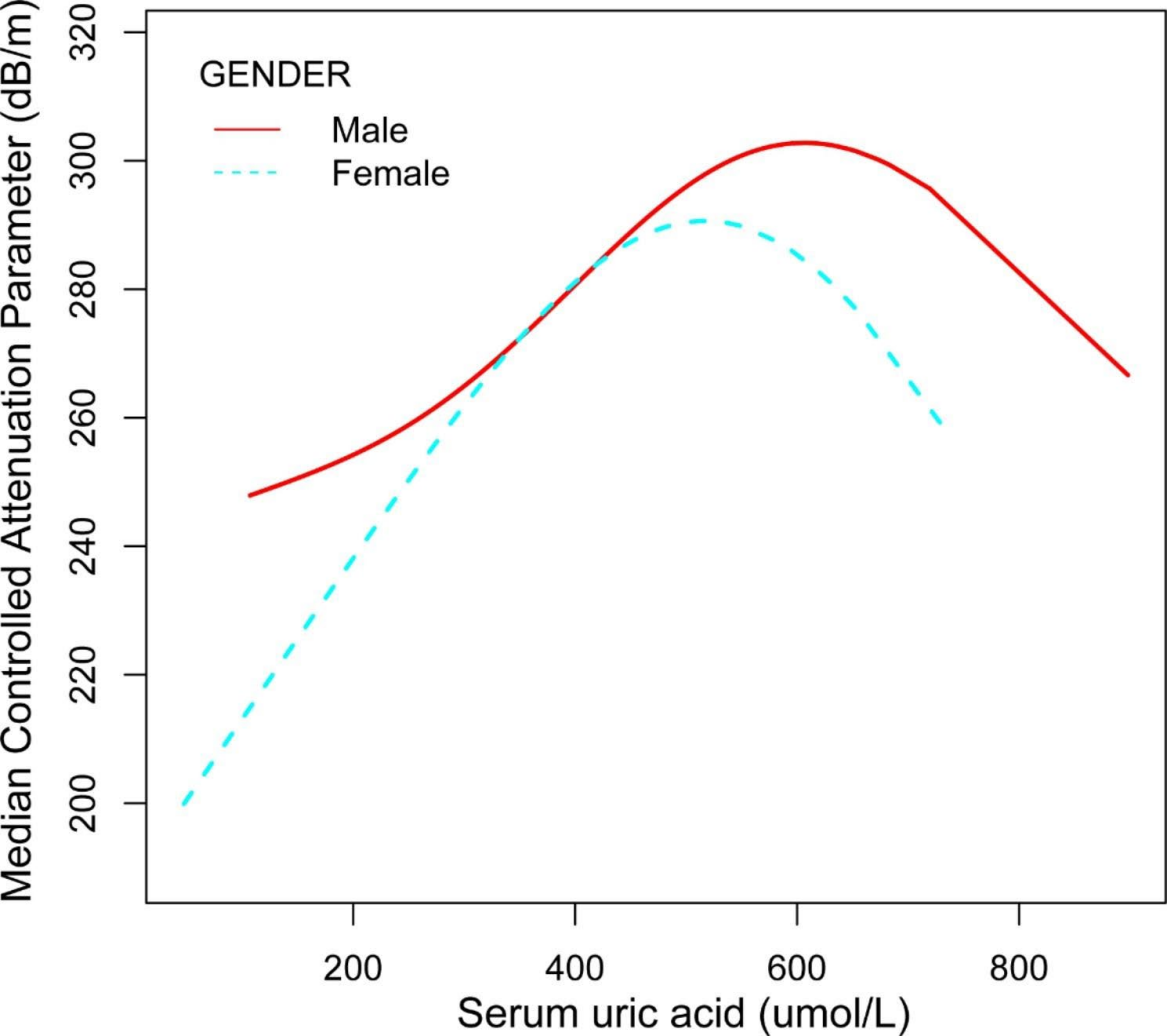
Association between SUA and CAP among US adults. The solid red line represents the relationship in males, and the blue bars represent the relationship in females. Adjust for age, race, BMI, waist circumference, education levels, ratio of family income, ALT, AST, GGT, serum creatinine, triglyceride, serum cholesterol, HDL-C, LDL-C, glycohemoglobin, HOMA-IR, dietary fat intake, smoking habits, MET, hypertension and diabetes

Smooth curve fitting revealed a nonlinear correlation between SUA and CAP (Fig. 1). The threshold effect of SUA on CAP is observed in Table 3. A significant association between SUA and CAP existed inside the inflection point (SUA = 475.8 µmol/L) (β = 0.18, 95% CI: 0.16-0.21, *P* < 0.01). There was no connection after the inflection (β = −0.01, 95% CI: −0.06-0.04, *P* = 0.64). Similar relationships were obtained for males and females; the inflection points were 487.7 µmol/L and 386.6 µmol/L, respectively. The relationship between SUA and CAP existed as an inflection point for both males and females.

**Table 3:** Threshold effect analysis of SUA (µmol/L) on CAP using the segmented regression model

	Total	*P* values	Male	*P* values	Female	*P* values
The standard linear model	0.16 (0.14, 0.19)	< 0.01	0.14 (0.11, 0.18)	< 0.01	0.19 (0.16, 0.23)	< 0.01
The segmented model						
Inflection points of SUA (µmol/L)	475.8		487.7		386.6	
< inflection point	0.18 (0.16, 0.21)	< 0.01	0.16 (0.13, 0.18)	< 0.01	0.24 (0.21, 0.27)	< 0.01
> inflection point	−0.01 (−0.06, 0.04)	0.64	0.03 (−0.04, 0.09)	0.43	0.01 (−0.04, 0.05)	0.82
Log likelihood ratio	< 0.01		< 0.01		< 0.01	

Adjust for age, race, BMI, waist circumference, education levels, ratio of family income, ALT, AST, GGT, serum creatinine, triglyceride, serum cholesterol, HDL-C, LDL-C, glycohemoglobin, HOMA-IR, dietary fat intake, smoking habits, MET, hypertension and diabetes

There was a positive association between SUA (mg/dL) and NAFLD, according to Table 4. Regardless of the optimum cutoff values for NAFLD (248 dB/m, 268 dB/m, or 280 dB/m), the positive associations were consistent in both males and females after multiple imputations. The correlations between SUA and NAFLD in males in Model 2 were insignificant before imputation. Stratified by race, as shown in Supplementary Table S2, positive relationships were also observed.

Hyperuricemia and NAFLD were found to be positively related (OR = 1.94, 95% CI: 1.64-2.30, *P* < 0.01), as shown in Table 5. Stratified by gender, the P for interaction was less than 0.01 in model 2, and the positive correlation was more significant in females than in males.

**Table 4:** Association between SUA (mg/dL) and NAFLD among US adults aged ≥ 20 years

	Crude	*P* values	Model 1	*P* values	Model 2	*P* values	Model 1^a^	*P* values	Model 2^a^	*P* values
CAP ≥ 248 (dB/m)										
SUA	1.40 (1.33, 1.48)	< 0.01	1.35 (1.28, 1.43)	< 0.01	1.29 (1.13, 1.47)	< 0.01	1.38 (1.31, 1.45)	< 0.01	1.35 (1.27, 1.42)	< 0.01
Male	1.29 (1.20, 1.38)	< 0.01	1.28 (1.18, 1.38)	< 0.01	1.16 (0.96, 1.39)	0.14	1.30 (1.21, 1.39)	< 0.01	1.28 (1.19, 1.38)	< 0.01
Female	1.55 (1.43, 1.68)	< 0.01	1.45 (1.33, 1.58)	< 0.01	1.55 (1.24, 1.93)	< 0.01	1.48 (1.37, 1.61)	< 0.01	1.43 (1.32, 1.56)	< 0.01
CAP ≥ 268 (dB/m)										
SUA	1.35 (1.28, 1.42)	< 0.01	1.31 (1.24, 1.38)	< 0.01	1.30 (1.14, 1.47)	< 0.01	1.33 (1.26, 1.40)	< 0.01	1.30 (1.23, 1.37)	< 0.01
Male	1.23 (1.15, 1.32)	< 0.01	1.23 (1.14, 1.32)	< 0.01	1.10 (0.92, 1.30)	0.18	1.24 (1.16, 1.32)	< 0.01	1.22 (1.14, 1.31)	< 0.01
Female	1.50 (1.39, 1.62)	< 0.01	1.42 (1.31, 1.54)	< 0.01	1.71 (1.36, 2.15)	< 0.01	1.45 (1.34, 1.57)	< 0.01	1.40 (1.29, 1.51)	< 0.01
CAP ≥ 280 (dB/m)										
SUA	1.33 (1.27, 1.40)	< 0.01	1.30 (1.24, 1.38)	< 0.01	1.27 (1.12, 1.43)	< 0.01	1.31 (1.25, 1.38)	< 0.01	1.28 (1.22, 1.35)	< 0.01
Male	1.24 (1.16, 1.32)	< 0.01	1.24 (1.15, 1.33)	< 0.01	1.08 (0.91, 1.28)	0.19	1.24 (1.16, 1.33)	< 0.01	1.23 (1.15, 1.32)	< 0.01
Female	1.45 (1.35, 1.56)	< 0.01	1.39 (1.28, 1.51)	< 0.01	1.66 (1.32, 2.09)	< 0.01	1.40 (1.30, 1.51)	< 0.01	1.35 (1.25, 1.46)	< 0.01

^a^ Missing values of covariates were imputed with multiple imputations

Model 1: age, race and BMI were adjusted

Model 2: age, race, BMI, waist circumference, education levels, ratio of family income, ALT, AST, GGT, serum creatinine, triglyceride, serum cholesterol, HDL-C, LDL-C, glycohemoglobin, HOMA-IR, dietary fat intake, smoking habits, MET, hypertension and diabetes were adjusted

**Table 5:** Association between hyperuricemia and NAFLD among US adults aged ≥ 20 years

	Crude	*P* values	Model 1	*P* values	Model 2	*P* values	Model 1^a^	*P* values	Model 2^a^			*P* values
Hyperuricemia	2.18 (1.85, 2.58)	< 0.01	2.16 (1.80, 2.58)	< 0.01	2.10 (1.35, 3.28)	< 0.01	2.10 (1.77, 2.48)	< 0.01	1.94 (1.64, 2.30)			< 0.01
Stratified by gender												
Male	1.78 (1.42, 2.22)	< 0.01	1.91 (1.50, 2.43)	< 0.01	1.30 (0.70, 2.44)	0.31	1.79 (1.43, 2.24)	< 0.01	1.72 (1.37, 2.17)			< 0.01
Female	2.79 (2.17, 3.57)	< 0.01	2.45 (1.87, 3.21)	< 0.01	4.18 (1.99, 8.76)	< 0.01	2.51 (1.95, 3.24)	< 0.01	2.24 (1.72, 2.90)			< 0.01
*P* for interaction										<0.01		

^a^ Missing values of covariates were imputed with multiple imputations

Model 1: age, race and BMI were adjusted

Model 2: age, race, BMI, waist circumference, education levels, ratio of family income, ALT, AST, GGT, serum creatinine, triglyceride, serum cholesterol, HDL-C, LDL-C, glycohemoglobin, HOMA-IR, dietary fat intake, smoking habits, MET, hypertension and diabetes were adjusted

## Discussion

In our study, we explored the association of serum uric acid with hepatic steatosis detected by a controlled attenuation parameter in the US population. There was a positive relationship between SUA and CAP, as well as between SUA and NAFLD, in both males and females. After stratification by race, positive relationships were also observed. The relationship between SUA and CAP existed as an inflection point for both males and females. Within the inflection point, a positive relationship existed; beyond the infection point, there was no relationship.

The primary results of our investigation are broadly similar to previous findings. However, gender differences in the relationship between SUA and NAFLD remain debatable. Previous meta-analyses showed that young women were at higher risk than age-matched men and older women [20]. Wang et al. found that the correlation between SUA and NAFLD existed in women but not in men [18]. Their results could be attributed to missing values when compared to the findings of our investigation. Meanwhile, missing data are a prevalent issue with publicly available datasets. In our study, there were missing variables, which we addressed with multiple imputations. Multiple imputations are a technique for reducing missing values and providing a trustworthy dataset for study [21]. We found that the associations were consistent among different sexes after the application of multiple imputations. Furthermore, we observed associations between hyperuricemia and NAFLD. The positive correlation was more significant in females than in males in the interaction test. The findings of a study of 166 individuals with biopsy-proven NAFLD were identical to ours, and females were at higher risk. [16]. Based on the study population, these association studies were most regularly reported in East Asian populations, particularly in China [22, 23]. The multiracial and multicultural nation’s NHANES database shows a representatively larger population. The previous studies did not evaluate ethnic subgroups. Therefore, we conducted an ethnicity-based subgroup analysis, and the findings in all racial subgroups were consistent. Gender and ethnicity had no effect on the observed relationships in this study.

The controlled attenuation parameter (CAP) assessed by the FibroScan device, which is a marker of hepatic steatosis, can be derived by simultaneously monitoring the attenuation of the ultrasound signal via the liver. In noninvasive examinations, B-mode ultrasonography, the most common method for detecting steatosis, is unreliable in assessing steatosis severity, especially in mild cases [24]. The magnetic resonance imaging (MRI) technique is extremely precise in detecting and quantifying HS [25], but it is insufficiently available to analyze a large sample of patients [11]. The FibroScan device is the most acceptable for the patient, but it has a variety of computed cutoff values [26]. HS, according to histological findings, is classified as absent-S0 (normal liver), mild-S1, 5–33%, moderate-S2, 36–66%, and severe-S3, more than 66%. In a meta-analysis [27], the overall performance of CAP compared to hepatic biopsy was as follows: S1 had a cutoff value of 248 dB/m (AUROC 0.82); S2, 268 dB/m (AUROC 0.86); and S3, 280 dB/m (AUROC 0.88). Cutoff values of 248 dB/m, 268 dB/m, and 280 dB/m were used in our study as sensitivity analyses. The sensitivity analyses revealed no significant changes in the results, indicating that the results were stable.

In this study, an inflection point is first reported between SUA and CAP in both males and females. Liver biopsy evaluation is impractical in a large sample population. Because CAP correlates well with histological steatosis [28], there may be a nonlinear relationship between SUA and HS. The inflection point was 487.7 µmol/L in males and 386.6 µmol/L in females. The significance of the inflection point is unknown, and we have no answer for this problem; additional research is needed. However, we can hypothesize that when the urate concentration exceeds 405 µmol/L, the limit of solubility under physiological conditions, urate crystals may form as monosodium urate (MSU). Because urate has both anti-inflammatory and proinflammatory effects, uric acid has the most impact on NAFLD at the inflection point [29].

Despite numerous studies, the underlying mechanisms between SUA and NAFLD remain unclear. Although SUA is positively associated with related metabolic disorders, it does not appear to be a causative factor. In the Mendelian randomized study, we still lacked evidence of pathogenicity between SUA and NAFLD [30]. In animal research, studies have shown that inhibition of xanthine oxidase may reduce uric acid production and decrease liver fat accumulation [31, 32]. However, there was research that supported the opposite viewpoint: inhibition of xanthine oxidase reduces uric acid but does not influence metabolic homeostasis [33].

### Study strengths and limitations

Notably, the major strengths of this study should be considered. First, this is a large-sample study using a large population (3919 participants) drawn from the NHANES database and using high-precision VCTE compared with ultrasonography. Second, in our research, multiple imputation, the most accurate method of imputation, was used to solve missing values. Sensitivity analysis and subgroup analysis were performed to show consistent results. Nonetheless, there were constraints. First, owing to the cross-sectional nature of the investigation, a causal link cannot be established. Second, we controlled as many covariates as possible; additional covariates such as drug use and period status might have caused bias. Third, we are aware of the missing hepatic biopsy. However, in large-sample investigations, biopsies are both impractical and unethical.

## Conclusion

There was a positive association between SUA and CAP, as well as between SUA and NAFLD. Subgroup studies stratified by sex and ethnicity demonstrated that the effects were consistent. However, further mechanistic study is still needed.

## Electronic supplementary material

Below is the link to the electronic supplementary material.

Supplementary Material 1

## Data Availability

The database used for this investigation can be found in online repositories. For more information, visit https://www.cdc.gov/nchs/nhanes/index.htm.
