# Supplementary material for: Association of serum uric acid with hepatic steatosis detected by controlled attenuation parameter in the United States population
Source: Lipids Health Dis. 2023 Jun 20;22:76. doi: 10.1186/s12944-023-01846-8 (PMC10280954; doi:10.1186/s12944-023-01846-8)
Supplement: Supplementary file 1 — Supplementary Material 1 [file 12944_2023_1846_MOESM1_ESM.docx]

Supplementary files

Table S1. Association between SUA (umol/L) and CAP among US adults aged ≥ 20 years

|  | Non-Hispanic White | *P* values | Non-Hispanic Black | *P* values | Mexican American | *P* values | Other Hispanic | *P* values | Other Race | *P* values |
| --- | --- | --- | --- | --- | --- | --- | --- | --- | --- | --- |
| Crude | 0.19 (0.15, 0.22) | <0.01 | 0.16 (0.12, 0.21) | <0.01 | 0.16 (0.11, 0.22) | <0.01 | 0.23 (0.16, 0.30) | <0.01 | 0.21 (0.16, 0.25) | <0.01 |
| Model 1^a^ | 0.16 (0.12, 0.20) | <0.01 | 0.15 (0.10, 0.20) | <0.01 | 0.11 (0.05, 0.17) | <0.01 | 0.22 (0.14, 0.29) | <0.01 | 0.19 (0.14, 0.25) | <0.01 |
| Model 2^a^ | 0.16 (0.12, 0.20) | <0.01 | 0.14 (0.09, 0.19) | <0.01 | 0.11 (0.05, 0.17) | <0.01 | 0.21 (0.14, 0.29) | <0.01 | 0.20 (0.15, 0.25) | <0.01 |

^a^ Missing values of covariates were imputed with multiple imputation.

Model 1: age, gender and BMI were adjusted

Model 2: age, gender, BMI, waist circumference, education levels, ratio of family income, ALT, AST, GGT, serum creatinine, triglyceride, serum cholesterol, HDL-C, LDL-C, glycohemoglobin, HOMA-IR, dietary fat intake, smoking habits, MET, hypertension and diabetes were adjusted.

Table S2. Association between SUA (mg/dL) and NAFLD (CAP≥268 dB/m) among US adults aged ≥ 20 years

|  | Non-Hispanic White | *P* values | Non-Hispanic Black | *P* values | Mexican American | *P* values | Other Hispanic | *P* values | Other Race | *P* values |
| --- | --- | --- | --- | --- | --- | --- | --- | --- | --- | --- |
| Crude | 1.40 (1.29, 1.51) | <0.01 | 1.31 (1.19, 1.43) | <0.01 | 1.28 (1.14, 1.44) | <0.01 | 1.45 (1.24, 1.70) | <0.01 | 1.42 (1.28, 1.58) | <0.01 |
| Model 1^a^ | 1.35 (1.24, 1.48) | <0.01 | 1.28 (1.15, 1.42) | <0.01 | 1.23 (1.08, 1.41) | <0.01 | 1.45 (1.22, 1.72) | <0.01 | 1.38 (1.23, 1.56) | <0.01 |
| Model 2^a^ | 1.36 (1.25, 1.49) | <0.01 | 1.27 (1.14, 1.41) | <0.01 | 1.22 (1.08, 1.41) | <0.01 | 1.44 (1.19, 1.72) | <0.01 | 1.42 (1.26, 1.60) | <0.01 |

^a^ Missing values of covariates were imputed with multiple imputation.

Model 1: age, gender and BMI were adjusted

Model 2: age, gender, BMI, waist circumference, education levels, ratio of family income, ALT, AST, GGT, serum creatinine, triglyceride, serum cholesterol, HDL-C, LDL-C, glycohemoglobin, HOMA-IR, dietary fat intake, smoking habits, MET, hypertension and diabetes were adjusted.
